# An alternative to jail by assertive community treatment and housing for people with severe psychiatric disorders and insecure accommodation: a potential win-win outcome of reduced recidivism and reduced public expenditure

**DOI:** 10.1192/j.eurpsy.2022.1959

**Published:** 2022-09-01

**Authors:** S. Loubiere, A. Tinland, T. Bosetti, P. Auquier

**Affiliations:** 1 APHM, Service Epidemiologie And économie De La Santé, Direction De La Recherche, MARSEILLE, France; 2 APHM, Marss, Marseille, France; 3 Medecins du Monde, Ailsi, Marseille, France

**Keywords:** Social Impact Contract, Incarceration, housing insecurity, Severe Mental Disorders

## Abstract

**Introduction:**

People with severe psychiatric disorders (SPD) who experience housing vulnerability have to negotiate discontinuous mental health care pathways including poor access to common rights services and an increased risk of incarceration. To reduce morbidity and improve social integration of these people, Médecins du Monde (NGO), in association with the Ministry of Justice and APHM, is piloting the experimentation of an alternative to prison through assertive community treatment (ACT) and independent housing for people with SPS without housing who are referred to the court for immediate appearance.

**Objectives:**

The main objective is to evaluate the effectiveness of the innovative program (AILSI) compared to usual services by assessing the duration of re-incarceration at 18 months of follow up.

**Methods:**

The AILSI project has been certified as a Social Impact Bond, in which private investors support the program, with the guarantee that the French government will reimburse the investments if social impact outcomes are met. To measure the effectiveness and efficiency of the program, a randomized controlled study was designed: 100 patients will be included in the AILSI group (intervention) and 120 in the TAU group (usual services). Four social impact outcomes are identified: inclusion rate, signed leases rate, total length of re-incarceration and total resource use. It is a mixed quali-quanti research, which integrates a matching to administrative health and judicial databases.

**Results:**

Inclusions are ongoing.

**Conclusions:**

The AILSI program and the research methods used are described herein. In addition, detailed information on the limitations and strengths of the SIB system are also discussed.

**Disclosure:**

No significant relationships.

